# Primary Bilateral Non-Hodgkin's Lymphoma of the Adrenal Gland: A Case Report

**DOI:** 10.1155/2012/325675

**Published:** 2012-12-10

**Authors:** Ahmed Amine Bouchikhi, Mohamed fadl Tazi, Driss Amiroune, Soufiane Mellas, Jalaledine El Ammari, Abdelhak Khallouk, Mohammed Jamal El fassi, Moulay Hassan Farih

**Affiliations:** ^1^Department of Urology, University Hospital of Fez, 30000 Fez, Morocco; ^2^Rue Zag, Rce Andalous III, Quarier Al-Wafe, 30070 Fes, Morocco; ^3^Anatomy Laboratory, Faculty of Medicine and Pharmacy, University of Fez, 30000 Fez, Morocco

## Abstract

Primary bilateral non-Hodgkin's lymphoma (NHL) of the adrenal gland is a very rare entity. Indeed less than 60 cases have been reported in the literature. Hence, we report a case of high-grade lymphoma of both adrenal glands that was found in a young patient of 32 years of age. The patient was admitted in the emergency department of our hospital with a profile of hemorrhagic shock. After stabilization, the imaging investigations demonstrated large bilateral adrenal masses. The CT-scan guided biopsy of both adrenal glands allowed the diagnosis of primary bilateral adrenal NHL. The patient died after the first chemotherapy session. The presence of bilateral adrenal masses associated with a rapid increase of volume should raise the diagnosis of primary adrenal non-Hodgkin's lymphoma.

## 1. Introduction

The adrenal mass has a significant etiological diagnosis problem. Thus, primary lymphoma of the adrenal gland is a rare origin of adrenal tumors; it has to be evoked specifically whenever bilateral adrenal affections are revealed. We report a case of a primary bilateral adrenal lymphoma. We discuss the clinical feature and paraclinical and therapeutic issues with literature review [1–4].

## 2. Case Report

The patient is a 32-year-old married lady. The patient was admitted in the emergency department of our hospital with a profile a hemorrhagic shock. The interview revealed a patient with bilateral back pain of 5 months history without irradiation.

The general examination found a patient of poor general state with difficulties to measure blood pressure and showing a frank discolored connective. 

The patient followed an urgent blood filling. The abdominal examination found painful masses in both sides which were difficult to delineate without any hepatomegaly or splenomegaly. Neither signs of hyperadrenocorticism nor lymphadenopathy were found. The clinical examination of a primary neoplasm was negative.

**Figure 1:**
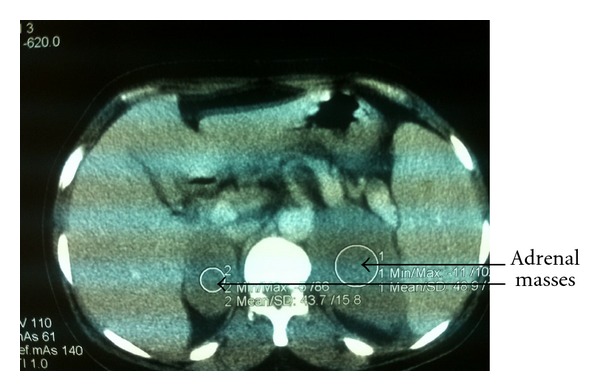
The abdominal CT scan demonstrated frankly bilateral volumes of adrenal masses.

**Figure 2:**
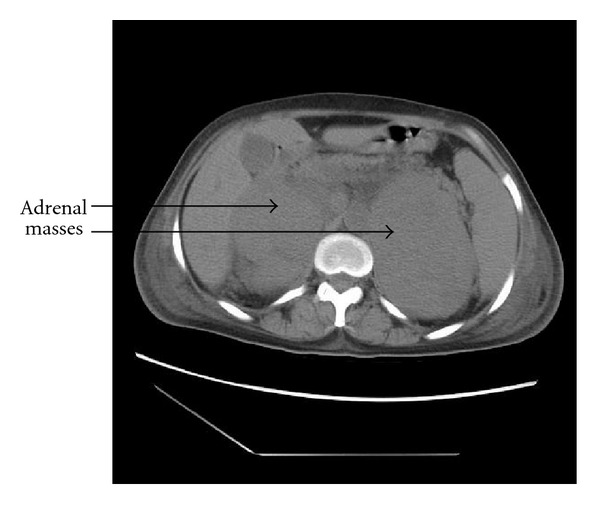
The abdominal CT scan showed an increase of the volume of adrenal masses.

**Figure 3:**
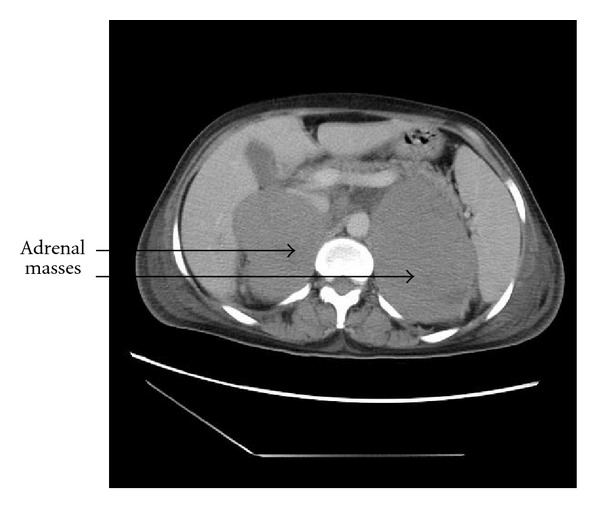
A slight contrast enhancement of the CT scan demonstrated an increase of the volume of adrenal masses.

The biological assessment of the adrenal hormones was normal. The chest radiography was without abnormality. The abdominal ultrasound revealed bilateral adrenal masses, while the abdominal CT scan objectified bilateral adrenal masses of hypodense tissue measuring 7 cm and 10 cm diameter in the right and left sides, respectively (Figure 1). Poly-retro-peritoneal lymph nodes measuring 10 to 20 cm diameter meters were also found. A second CT scan was achieved with contrast enhancement and demonstrated an increased size of the adrenal masses (Figures 2 and 3). A CT-scan guided biopsy was performed for diagnostic purposes. The histological examination showed a non-Hodgkin's lymphoma with large cells of B phenotype expressed by diffuse and intense membrane immunostaining with CD20, and absence of immunostaining with CD3.

During hospitalization, the patient state rapidly altered with weight loss of 10 kg per day; the patient died after the first session of chemotherapy.

## 3. Discussion

The primitive lymphomas are a rare cause of adrenal tumors. Less than 70 cases were reported in the literature [5–8]. Men are the most affected with an age interval between 39 and 89 years with a mean 68 years of age. Our case is found out of the age range assessed so far since this patient is a woman 32 years old.

Mostly, the affection is bilateral with impaired adrenal glands and adrenal function and such was our case.

The etiopathogenesis is still unknown. However frequent association with autoimmune diseases is assessed [9–11].

Clinically, this tumor is expressed by abdominal pain in 26% of cases, unexplained fever in 46% of patients, and impaired general health with weight loss connected with lymphoma in 24% of patients. Adrenal deficiency might occur with manifestations of skin pigmentation and orthostatic hypotension. Our patient was admitted with a profile of altered general state.

The primitive adrenal NHL involves differential diagnosis problems with other adrenal benign tumors such as pheochromocytoma, hematoma, infection, and adenoma or malignant tumors such adrenocortical carcinoma and metastases.

Recently, Kumar et al. used SPECT by fluorodeoxyglucose scan in the evaluation and monitoring of a patient with primary adrenal NHL [12].

The diagnosis is initially directed by imaging approaches including adrenal ultrasound and CT scan that are generally showing a homogeneous oval and well circumscribed mass, slightly enhanced by the contrast agent [13].

The final diagnosis is demonstrated by histological arguments using ultrasound-guided or CT-scan guided biopsy with immunohistochemical study [1].

Our patient underwent a biopsy CT-scan guided biopsy that demonstrated a large NHL cell of B phenotype. This phenotype is predominant (9/11 cases tested) according to Ambroziak et al. [13], Dutta et al. [14], Horiguchi et al. [15].

The treatment of this rare disease consists of chemotherapy with or without radiotherapy. It is to note that surgery is not recommended. The prognosis remains very poor with recession within six months.

## 4. Conclusion

Primary bilateral non-Hodgkin's lymphoma of the adrenal gland is very rare entity. It affects mainly men but women are not excluded. Primary bilateral non-Hodgkin's lymphoma of the adrenal gland has to be evoked whenever bilateral adrenal masses are assessed in the CT-scan images. The diagnosis is essentially histological. The chemotherapy with or without radiotherapy is the usual treatment. The prognosis remains poor.
